# Smart Underwater Sensor Network GPRS Architecture for Marine Environments

**DOI:** 10.3390/s25113439

**Published:** 2025-05-30

**Authors:** Blanca Esther Carvajal-Gámez, Uriel Cedeño-Antunez, Abigail Elizabeth Pallares-Calvo

**Affiliations:** Instituto Politécnico Nacional, Unidad Profesional Interdisciplinaria en Ingeniería y Tecnologías Avanza-das, Mexico City 07340, Mexico; ucedenoa1200@egresado.ipn.mx (U.C.-A.); apallaresc@ipn.mx (A.E.P.-C.)

**Keywords:** marine sensor network, internet of things, mobile architecture, wireless communication

## Abstract

The rise of the Internet of Things (IoT) has made it possible to explore different types of communication, such as underwater IoT (UIoT). This new paradigm allows the interconnection of ships, boats, coasts, objects in the sea, cameras, and animals that require constant monitoring. The use of sensors for environmental monitoring, tracking marine fauna and flora, and monitoring the health of aquifers requires the integration of heterogeneous technologies as well as wireless communication technologies. Aquatic mobile sensor nodes face various limitations, such as bandwidth, propagation distance, and data transmission delay issues. Owing to their versatility, wireless sensor networks support remote monitoring and surveillance. In this work, an architecture for a general packet radio service (GPRS) wireless sensor network is presented. The network is used to monitor the geographic position over the coastal area of the Gulf of Mexico. The proposed architecture integrates cellular technology and some ad hoc network configurations in a single device such that coverage is improved without significantly affecting the energy consumption, as shown in the results. The network coverage and energy consumption are evaluated by analyzing the attenuation in a proposed channel model and the autonomy of the electronic system, respectively.

## 1. Introduction

Seventy-five percent of the Earth’s surface is water, encompassing seas, rivers, and lakes [1]; these areas are inhabited by animals and aquatic vegetation, among other types of life. In recent years, the temperature of marine waters has increased due to current climate change, the greenhouse effect, and toxic waste derived from various human activities, such as tourism, industry, and urban development [2].

Ecologists, biologists, technologists, scientists, and researchers have proposed and implemented techniques and mechanisms for monitoring aquatic habitats for conservation purposes and monitoring areas at risk of contamination and overexploitation; notably, studies of nutrient levels and beach erosion have been performed [3], such as in the analyses of Sargassum along the Mexican coast, which has affected the economy and ecology of the area. The IoT involves communication technologies such as Bluetooth (BT), radio frequencies (RFs), cellular communication, satellite communication, and general packet radio services (GPRSs), among others.

Mobile sensor networks are considered high-potential tools because of their low cost, high portability, and easy handling, unlike other monitoring mechanisms, which can be more complex and expensive [2]. To establish connections and interconnections among nodes, sensors, and remote stations in the aquatic environment, the underwater Internet of Things (UIoT) was constructed. In addition, the development of underwater wireless sensor networks (UWSNs) has led to the establishment of new criteria related to the following: (i) water resistance; (ii) robustness to environmental conditions; (iii) energy consumption; (iv) line of sight for communications; and (v) others [1].

UWSNs fuse two technologies, namely, wireless and small sensors, the latter with intelligent sensing and computing capabilities and the ability to establish effective communication. A UWSN is an autonomous network of sensor nodes that are spatially distributed under or over water to detect certain characteristics related to the environment [1].

## 2. Related Work

This section summarizes the different works related to the design of aquatic mobile sensor networks and the different marine communication mechanisms currently available.

### 2.1. Underwater Sensor Networks

The development of network architecture and components directly depends on the particular needs for which they are to be implemented [4]. One of the main challenges in the development of mobile sensor networks is optimizing their adaptability to the environment, the reconfiguration of nodes, the scope of data transmission, energy consumption, and cost, which must be considered in the design phase. Different authors have proposed different architectures for UWSNs [1,5,6].

### 2.2. UWSN Marine Systems

Researchers have begun to focus on projects, systems, and applications for the monitoring and surveillance of marine environments.

The authors of [7] developed smart environmental monitoring and analysis technology (SEMAT) for marine research and water monitoring. SEMAT comprises plug-and-play smart sensors, short-range wireless transmission, and near-real-time analysis tools. SEMAT can be used for quick and short-term deployments, especially in shallow coastal aquatic environments.

In [8], the authors proposed a cognitive network that uses optical communication with a transmission speed of 10 Mbps.

Another study [9] reported the results obtained in the implementation of a UWSN. In this network, they implemented communication between the nodes, with the support of the transmission delay allocation protocol MAC (TDA-MAC), for data collection in underwater acoustic sensor networks (UASNs).

In Ref. [10], the authors proposed a hybrid network that combines optical and acoustic communication. The results obtained via simulation were presented at distances of 30, 60, and 90 m. The average results of 1000 tests were presented for a transmission speed of 0.96 Mbps at 90 m as the worst case and 2.87 Mbps at 30 m as the best case.

Additionally, in various parts of the world, researchers have focused on integrating technology and implementing it in UWSNs, an example of which is the European Union project entitled Copernicus Marine Service [11], in which information collected from a UWSN is applied for monitoring the health of the ocean.

In Ref. [12], data transmission was performed with two NRF Arduino communication modules submerged in a pool at 25 cm, transmitting at two frequencies. When a 2.4 GHz frequency was implemented, a low delay of up to 40 cm was observed, and when a frequency of 433 MHz was used, the end-to-end delay remained stable up to 40 cm and then increased dramatically up to 90 cm, at which point the signal was no longer detected. The performance of the two modules is related to the distance in a water-to-water scenario. When 2.4 GHz is used, the performance is good at first but begins to degrade up to 40 cm, at which point the signal is lost. This is due to the high operating frequency of the NRF module, which allows nodes to send data at a higher rate when the attenuation is low than when it is high, i.e., when the distance between the nodes is very small. On the other hand, the performance is very poor at 433 MHz. However, the signal of the system is not lost after a short distance.

### 2.3. Wireless Communication in the Ocean

Currently, there are four technologies for underwater wireless communication. (1) RF technology is used for terrestrial wireless communication and underwater communications; it reaches high data transmission speeds in short communication, the disadvantage of which is the Doppler effect. (2) Optical communication is also used in marine environments where a blue–green wavelength is recommended for transmission over line-of-sight distances. (3) Magnetic induction technology, which is used for the UIoT, enables real-time communication at high bandwidths, as it is independent of environmental deterioration effects, such as multipath fading and time-varying signal distortion. (4) Finally, the most commonly used communication mechanism is acoustic communication due to the long communication range [13], which is limited by water salinity, turbidity, and temperature [14].

### 2.4. Characteristics of Each Mode of Wireless Communication

For acoustic communication, variations in environmental conditions can negatively influence the propagation of data, such as via wave spreading, high latency, or Doppler effects. Optical communication is used in underwater communication because of the scope and distance that can be achieved between two nodes. This type of communication is generally applied through commercial LEDs, as shown in [15], and depends on the directionality of the LEDs, the wavelength used when transmitting the data, and the type of medium in which it is transmitted. In Refs. [15,16], the main obstacles, advantages, and disadvantages of this type of communication are shown and compared with those of acoustic communication and RF. Wireless communication between nodes through magnetic induction (MI) is an emerging and promising alternative to traditional methods, and such communication methods have attracted significant attention in the past decade. MI communication is based on Faraday’s electromagnetic induction law, and several wired coils are used to exchange information [17]. Radiofrequency waves can be transmitted well in terrestrial settings. However, radio waves are severely attenuated in seawater. Ultra-low-frequency electromagnetic waves of 30 to 300 Hz can penetrate more than 100 m of seawater [18].

This work is focused on the Mexican coast, the architecture of the mobile sensor network there, and wireless communication via underwater and on-water media via the existing wireless communication technology. The design of an aquatic monitoring infrastructure system is generally associated with specific design and development requirements, such as noninvasiveness, low energy consumption, and economical and easily replicable communication. Therefore, the design of an architecture that uses wireless communication for the exchange of data between a remote center and a given object is proposed.

The main contributions of this work are as follows:

- A mobile sensor node for communication in marine environments is analyzed. The development of this type of technology provides connectivity in maritime zones, enabling monitoring of ocean and marine species health, fishing and tourist boat tracking, and tourist monitoring in activities considered to be of intermediate risk.
- A GPRS communication architecture is developed from a node to a ground base. As shown in the introduction and related works, there are works with other types of communication technology, such as RF, optical, and ultrasonic signals. The main intention of using this type of link is to start a communication and data transmission system that is friendly to the environment, that is, one that does not interfere with communication between species. In addition, there is no need to purchase more technology because cellular communication is currently available at low cost.
- The architecture of mobile nodes is designed for communication in mesh networks in marine environments. Proposing a type of connectivity that links all nodes, as in this work, even presenting a negative scenario in which any node is not available, connectivity prevails between them. Unlike what is available in related works, communication is unidirectional, and in a case of occlusion, there will be no communication.
- A high-performance computing architecture with low energy consumption is established. From the elements selected for the architecture of the proposed node, characteristics such as low energy consumption and easy-to-understand and configure architecture are considered. As justified further on in the text. The remainder of this paper is organized as follows: Section 2 presents a review of the works related to the research topic. Section 3 presents the architecture proposed in this work. Section 4 presents the tests and results. Section 5 presents the discussion and conclusions.

## 3. Materials and Methods

In this section, the proposed architecture for monitoring and the methodologies used for the evaluation of the proposed architecture are introduced. Notably, the energy consumption and simulation results related to the coverage of wireless links are evaluated.

Network applications in marine environments must overcome challenges associated with environmental conditions and the application characteristics. Some of these challenges are listed in [14], and the following were considered for the development of the network in this study.

**Figure 1:**
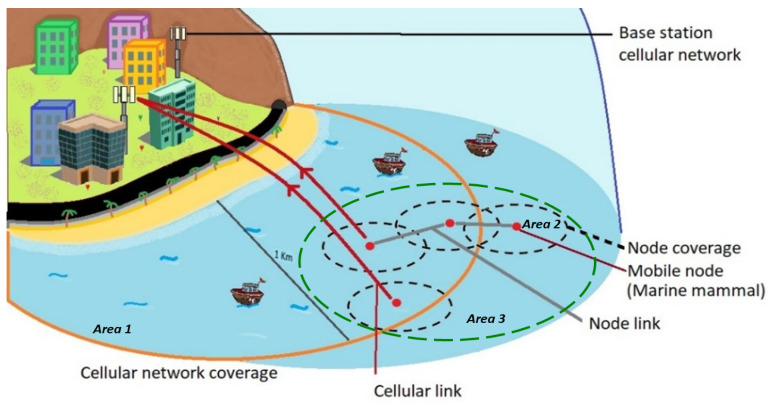
General architecture of the proposed implementation model.

(1) Water resistance: sensor nodes in a marine monitoring system require relatively high levels of water resistance, as they are constantly exposed to saturated conditions.
(2) Robustness: A marine monitoring system requires high robustness since the marine environment includes waves, sea currents, tides, typhoons, ship effects, etc., and is dynamic and complex; thus, nodes are constantly moving.
(3) Power consumption: power consumption is often high due to the long distance of transmission and various dynamic obstacles in the marine environment.
(4) There are several other issues that may be encountered, such as difficulty in deploying and maintaining nodes, the need for mooring devices and buoys, sensor coverage issues, and potential vandalism. In this work we consider points 2 and 3 in the analysis. Therefore, considering the above factors and the issues noted by Felenbam et al. [1], according to the types of architectures for UWSNs, the proposed architecture is a 2D-UWSN with a special exception, as our architecture does not require node clusters, and each node can act on its own. In Figure 1, we show the proposed implementation of the architecture.

We divide Figure 1 into different activity performance tasks.

Area 1 (the orange circle): This area corresponds to wireless link activities using cellular technology, in which there is a coverage of approximately 1 km radially. Within this area, information can be sent and received wirelessly using the 850 and 1900 MHz transmission bands for the GPRS and UMTS, respectively. UWSN’s computational architecture is designed to combine or switch between two generations of cellular technology to achieve the best quality for wireless links.

Area 2 (the black dotted circle): This area corresponds to the set of electronic systems for mobile nodes. These components are the GPS sensor, the wireless communication cards, and the information processing core.

Area 3 (the green dotted circle): This area corresponds to communication among the cluster nodes in the 433 MHz band, which are arranged in a mesh-type topology. Nodes within the cluster can act individually by connecting to the internet via cellular technology. Thus, information can be shared, and a node within the coverage of the cellular network can serve as a bridge to send information to other nodes in the cluster, acting as the head of the cluster momentarily.

**Figure 2:**
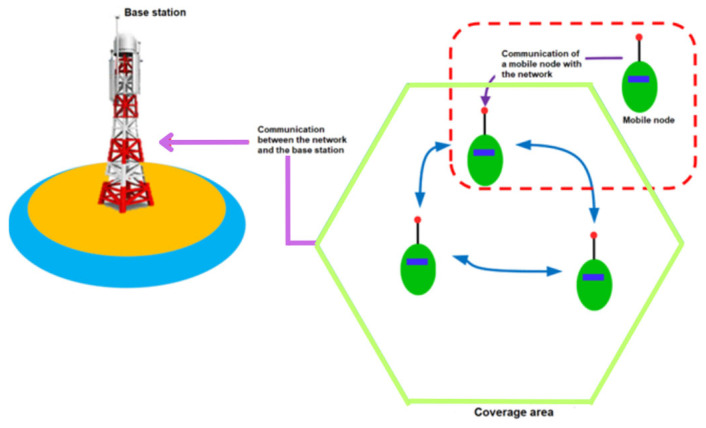
Network diagram of the proposed system.

More precisely, we present the specific architecture proposed as follows:

Figure 2 shows the factors that make up the proposed architecture. Related to Figure 1, we obtain the following: base station refers to the distribution antenna (Area 1); mobile node refers to any object that has a sensor node implemented (Area 2); and coverage area represents the communication range between nodes (Area 3).

**Figure 3:**
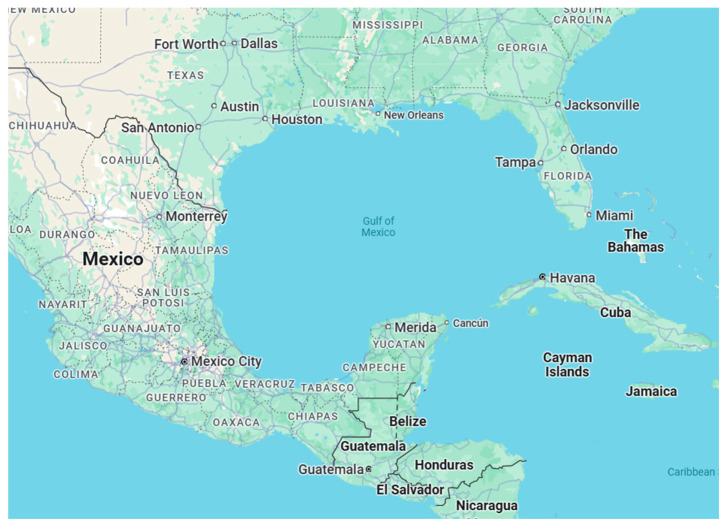
Map of the Gulf of Mexico [20].

In addition, it is important to consider the characteristics of the environment where the tests are to be carried out, for which the Gulf of Mexico (GoM) is the study area in this research, as shown in Figure 3. This basin is located in North America and has outlets to the Atlantic Ocean to the west and the Caribbean Sea to the south. This area is considered of low ocean depth, given that the maximum depth is 3000 m; for comparison, the Atlantic Ocean is approximately 8500 m deep [19].

**Figure 4:**
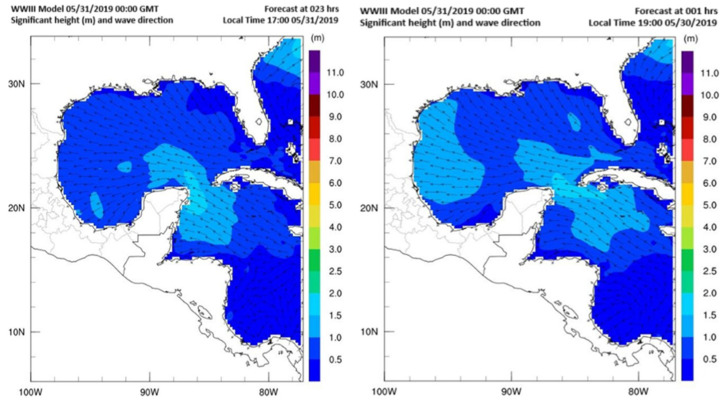
Wave maps for the GoM [22].

The GoM is characterized by low-amplitude waves of less than 0.5 m high [21] near continental masses and less than 1.5 m high near the outlets to the Caribbean Sea and Atlantic Ocean. In Figure 4, two maps of wave height in the GoM are shown, and the color scale corresponds to the maximum expected wave height, indicating that the waves in the Caribbean Sea are generally less than 2 m high. Therefore, the water parcels can be classified as calm ocean masses, except during certain disruptive events, such as cyclones and storms, among other phenomena [21].

### 3.1. Sensor Network Architecture

The requirements for the design of the sensor network architecture are as follows:

- The connection is maintained despite intermittent wireless communication.
- The wireless receiver is characterized by high sensitivity.
- There is a balance in the autonomy-portability relationship.

**Figure 5:**
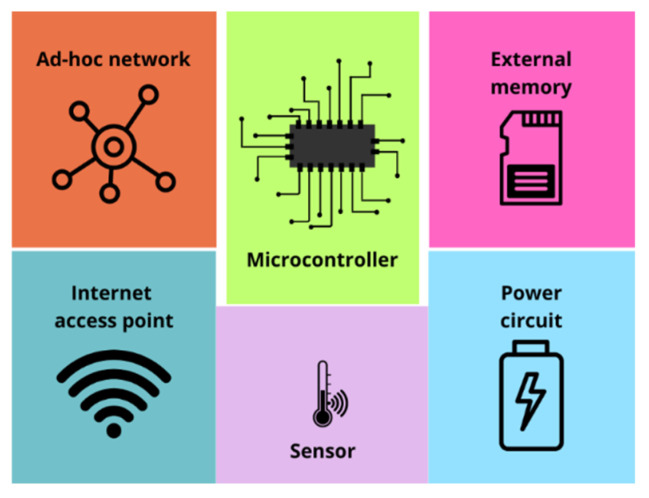
Diagram of the basic architecture of a network node.

Figure 5 illustrates the parts into which the embedded architecture of the WSN in this study is segmented.

**Figure 6:**
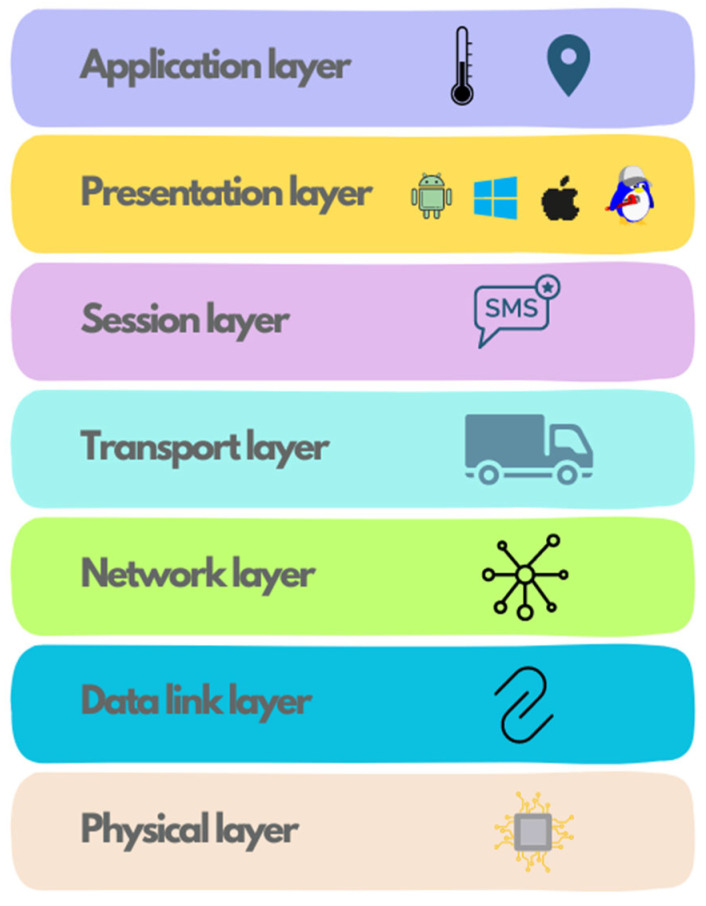
Architecture of the network of sensors for object monitoring.

Our proposed cognitive sensor network architecture not only optimizes real-time data collection and analysis but also improves the operational efficiency, decision-making, safety, and sustainability in a wide range of applications in marine environments. These advantages are illustrated in Figure 6, which shows a six-layer architecture with application, presentation, session, network, data link, and physical layers. The application layer encompasses the sensor, the memory, the power supply, which, in this case, is solar power, and the microcontroller, which is responsible for data collection. The presentation layer encompasses the operating system, which is the link between acquiring data from a sensor and sending the data to another node or cellular base. The session layer dictates what type of data transmission occurs. The transport layer is responsible for data flow control in the node network. The network layer supports connectivity and path selection for nodes. The data link layer ensures that data are transferred reliably from the sending node to the receiving node in a local network or between two directly connected nodes. The physical layer defines all the electrical and physical specifications for each node.

**Figure 7:**
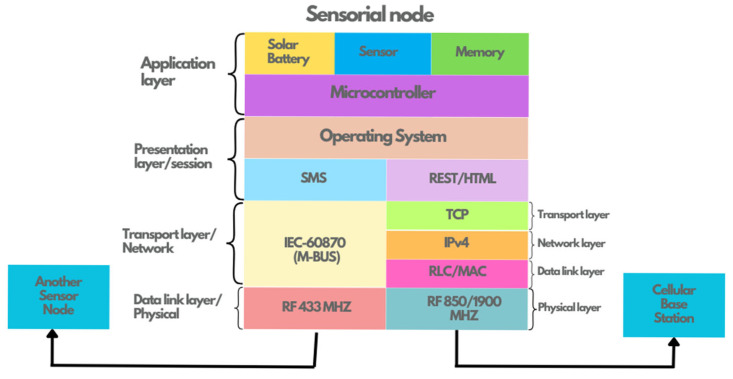
Internal description of the sensor node.

With these requirements, it is possible to make a breakdown of the requirements that the embedded system hardware must meet, and some of these are as follows: low-consumption electronic components, high sensitivity in wireless communication systems, and the incorporation of a removable memory. Figure 7 shows a diagram illustrating the parts into which the embedded architecture for the WSN of this work is segmented, in which it can be seen that in the center, there is a microcontroller as the central processing unit (CPU) of the system.

From Figure 7, the following description of the elements that make up the node is presented:

From Figure 7, we can observe in detail the elements that make up the internal composition by layer of the sensor node. Network protocols such as TCP/IP require a microcontroller to have large processing capabilities, and this condition led to the choice of this device being between 16- and 32-bit microcontrollers. Also, Figure 7 shows that RF is implemented at a frequency of 433 MHz for communication with another node, while RF at a frequency of 850/900 MHz is used for communication with the cellular base station. Table 1 shows a catalog of microcontroller options that were considered before the final choice.

The microcontroller that was selected is from the manufacturer STMicroelectronics, and it has the following characteristics: five synchronous or asynchronous serial communication ports, low consumption, and the lowest cost compared to the other options in Table 1.

#### Wireless Cards

The network structure of a WSN must be able to combine two different types of network topologies; in the particular case of this work, the combination of the star topology and the mesh topology is made. The star topology is used by cellular technology, and the mesh type is commonly implemented in ad hoc networks. The selected cellular network connection card is from the manufacturer Ublox, and its model is SARA u20; it has the following characteristics:

- Power supply: 3.3 V.
- Operating bands: 800, 850, 900, 1900, 2100.
- Sensitivity: −109 dBm with a BER < 2.04% (bits in error).
- Supports protocols: FTP, TCP/UDP.
- Operating modes: Normal, Sleep.
- IP support: IPv4, IPv6.
- Size: 2.6 × 1.6 × 0.3 (cm).
- Transmission power: 33 dBm.

To configure the ad hoc network, the wireless communication card must operate at the 433 MHz frequency and must also allow the ad hoc configuration of the WSN. The wireless card selected for the ad hoc network configuration is from the manufacturer Texas Instrument model CC1101.

### 3.2. Wireless Link

The wireless network links were evaluated via a wireless communication channel model. In this model, the effects of distortion of the wireless signals are related to the propagation distance and electrical properties of seawater and waves.

**Figure 8:**
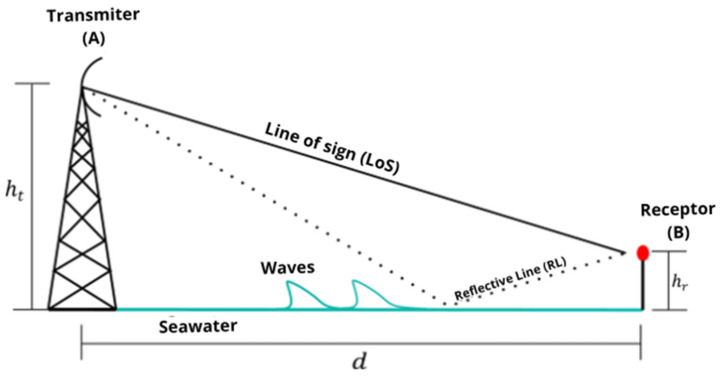
Scheme for the model approach. Source: the authors.

In Figure 8, a scheme for the channel model approach is shown, in which $h_{t}$ is the height of the transmission antenna, and $h_{r}$ is the height of the reception antenna; additionally, waves, line of sight (LoS), the reflected line (RL) are shown in relation to the seawater surface, and the red dot represents the sensor node.

The model uses Equation (1) for path loss, as shown below [23]:

(1) $${PL}_{dB}\left(d\right)=-((G_{t}G_{r}{\left(\frac{\lambda }{4\pi d}\right)}^{2}){\left|1+\Gamma e^{j∆\frac{4\pi h_{t}h_{r}}{\lambda d}}\right|}^{2})$$

where ${PL}_{dB}$ is the path loss, $G_{t}$ is the gain of the transmission antenna, $G_{r}$ is the gain of the receiving antenna, *λ* is the wavelength, *d* is the distance between the transmission antenna and the receiving antenna, *Γ* is the reflection coefficient, $h_{t}$ is the height of the transmission antenna, and $h_{r}$ is the height of the receiving antenna. The *Γ* reflection coefficient relates the power of the reflected signal to the power of the original signal, and the value of this coefficient is −1 for terrestrial transmission [24], which means that all incident power at the surface is reflected in the direction of the receiver. In marine environments, the electrical properties of seawater are dynamic due largely to the heterogeneity of salt concentrations [25]. A signal that hits the surface of seawater may not be fully reflected, so the value of this coefficient may fluctuate between the minimum (0) and the maximum value (−1). Notably, surface effects may significantly influence the attenuation of the signal at the receiver and contribute to other factors (absorption and transmission) that influence signal power. By modifying Equation (1) and considering the coefficient as an additional variable to the model, we obtain the following equation:

(2) $${PL}_{db}(d,t,\Gamma )=(G_{t}G_{r}{\left(\frac{\lambda }{4\pi d}\right)}^{2})({\left(1+\Gamma \right)}^{2}+{\left(\Gamma \frac{4\pi h_{t}h_{r}}{\lambda d}\right)}^{2})$$

The attenuation over the distance between (A) and (B) in Figure 6 can be obtained. This incident signal can be divided into two parts, one of which is reflected within the same medium by changing the propagation direction, while the other is transmitted to a second medium [26]. The magnitude of the signal power reaching the second medium can be obtained from the calculation of the transmission coefficient [27], which must comply with the following equation:

(3) $$\left|T\right|=1-\left|\Gamma \right|$$

where T is the transmission coefficient and is the ratio between the transmitted power and the incident power. The transmitted power is reflected in a “new” signal, which causes the signal to attenuate exponentially depending on these properties as a function of the absorption coefficient *α* [28]. Seawater is characterized as a medium for which electrical conductivity is low but not zero, so it can be classified as a dielectric with losses [28], which allows it to approximate α, as shown in the following equation:

(4) $$\alpha \approx \sqrt{f\mu \bar{\sigma }\pi }$$

where f is the frequency of the wireless signal, μ is the magnetic permeability of the propagation medium, and $\bar{\sigma }$ is the average electrical conductivity of the medium [26]. Furthermore, the point at which the signal hits the surface and is transmitted to the other medium can be considered a new “antenna”, so the transmitted signal is also attenuated as a function of distance; this attenuation is evaluated using the free space model in [29].

By combining all the phenomena involved in the transmission of a wireless signal in seawater and considering the fluctuation in the transmission coefficient due to the reflection coefficient, we can formulate the following path loss equation:

(5) $${{PL}_{dB}}_{T}\left(d,t,T\right)=-10\left((\left|T\right|)(e^{-\alpha d})\left(\frac{G_{r}G_{t}\lambda^{2}}{{\left(4\pi \right)}^{2}d^{2}}\right)\right)$$

Here, signal attenuation due to the electrical properties of the relevant media and the transmission distance are represented. In Equation (5), $G_{t}=1$ because the point of incidence is an ideal “antenna”. In the sea, waves can influence wireless links, producing effects, such as signal diffraction or dispersion, when signals are reflected at the surface of the water. Both phenomena affect the reception of information wirelessly. The magnitude of attenuation due to diffraction can be obtained by using the Kirchhoff parameter v [30], which is defined by the following equation:

(6) $$\nu =\sqrt{\frac{{h_{e}}^{2}2(d_{1}+d_{2})}{\lambda (d_{1}d_{2})}}$$

where $d_{1}$ is the distance between the transmitting antenna and a wave, $d_{2}$ is the distance between the receiving antenna and a wave, and $h_{e}$ is the height of the wave with respect to its intrusion into the Fresnel zone. This zone is defined as the ellipsoidal volume formed between the foci at the transmitting and receiving antennas [31].

From the parameter v, the following piecewise function is defined:

(7) $$K_{dB}\left(v\right)=\left\{\begin{array}{cc} \;\;\;\;\;\;\;\;\;\;\;\;\;0, & v<-1\;\\ \;\;\;\;\;\;\;\;20\;(0.5-0.62\nu ),\; & -1\leq v\leq 0\\ \;\;\;\;\;\;\;\;\;\;\;20\;(0.5e^{-0.95v}), & 0<v\leq 1\\ \;20\;(0.4-\sqrt{0.1184-\left(\;0.8-0.1v^{2}\right)}), & 1<v<2.4\;\\ 20\;(\frac{0.225}{v}), & 2.4<v \end{array}\right.$$

where $K_{dB}$ represents the attenuation due to diffraction produced by a wave.

W.S. Ammet [32] determined that the amount of the signal that is diffusely reflected is related to the reflection coefficient $\Gamma_{s}$, which is expressed via the following equation:

(8) $$\Gamma_{s}=\rho_{s}\Gamma_{v}$$

where $\rho_{s}$ is the diffuse reflected power density and is determined as follows:

(9) $$\rho_{s}=e^{-8{\left(\frac{\pi h_{c}sin\left(\theta_{i}\right)}{\lambda }\right)}^{2}}$$

where $h_{c}$ represents the height of the surface irregularities.

### 3.3. Embedded Computational Architecture

**Figure 9:**
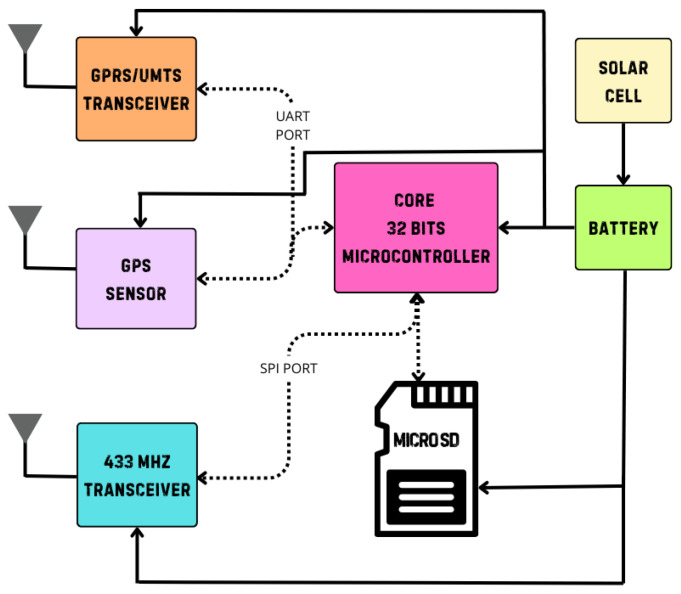
Diagram of mobile node architecture.

Network nodes can join a cluster by requesting permission or functioning as the cluster head. Specifically, each embedded system for each node must have qualities that allow it to manage digital batches of information from the internet, as well as communicate with other nodes in an ad hoc configuration. Therefore, a 32-bit microcontroller was selected as the processing component of the embedded system, and a diagram of the embedded system components is shown in Figure 9, in which the continuous arrows represent what each component is connected to, and the dotted arrows represent which ports are connected to the other component.

As shown in Figure 8, the proposed embedded system is composed of a microcontroller, which is a 32-bit system based on an ARM-RISC architecture, and a GPS, which allows the system to determine the geographic position of each mobile node. For communication, the system has a transceiver that is capable of operating at a frequency of 433 MHz. In addition, it has a GPRS/UTMS transceiver. Finally, the embedded system is powered by a lithium-ion battery with a current demand capacity of 3000 mAh and a nominal output voltage of 3.7 V. This battery recovers energy through a solar cell, which is a 1.1 W cell with a no-load output of 6 V.

## 4. Tests and Results

In this section, the tests of system components, the results of the simulations of the communication channel model, and tests of system hardware are introduced. Moreover, the expected network coverage is evaluated.

### 4.1. Testing of the Architecture

The architecture components were subjected to energy consumption tests to evaluate their performance and expected autonomy; a data acquisition system was used for this purpose.

### 4.2. Marine Wireless Communication Channel

**Figure 10:**
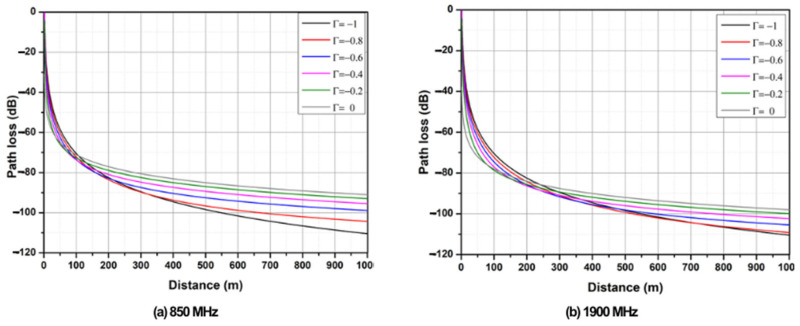
Path loss over seawater.

The frequencies that were evaluated were 433, 850, and 1900 MHz; the last two are central frequencies in the transmission bands for GPRS and UMTS technology, according to the laws and regulations in Mexico established by the Federal Telecommunications Institute [33]. By using Equation (2), which describes the attenuation of a transmitted signal, and numerical simulations, the attenuation behavior of a wireless signal in the marine environment was observed. In Figure 10a, path loss is observed at an 850 MHz frequency, whereas in Figure 10b, path loss is observed at a 1900 MHz frequency, with a maximum simulation distance of 1000 m, considering $h_{t}$ = 15 m, $h_{r}$ = 0.2 m, and ideal antenna gains.

**Figure 11:**
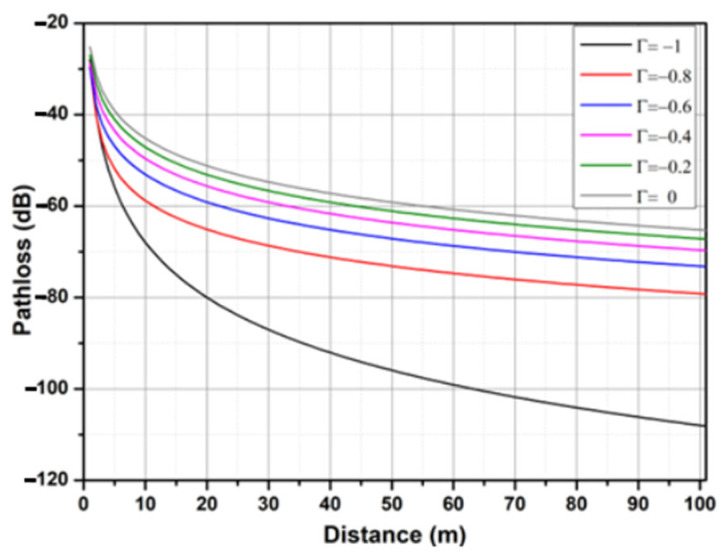
Path loss over seawater at the 433 MHz frequency.

The simulation of the attenuation on the seawater surface at 433 MHz is shown in Figure 11, where the maximum simulation distance is 100 m, $h_{t}$ = 0.2 m, $h_{r}$ = 0.2 m, and the antenna gains are considered ideal.

**Figure 12:**
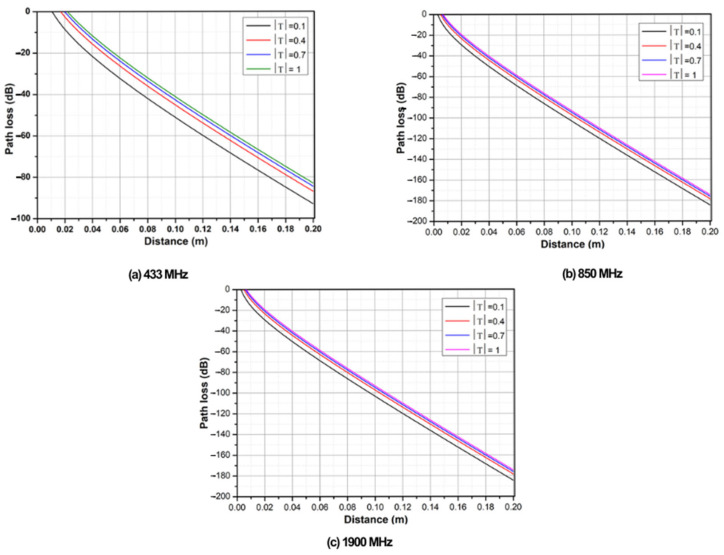
Path loss in seawater at (**a**) 433 MHz, (**b**) 850 MHz, and (**c**) 1900 MHz.

To evaluate the behavior of the signal below the seawater surface, a numerical simulation based on Equation (5) and Equation (4) was used, and α values of 82.69, 115.9, and 173.2 Np/m were obtained at frequencies of 433, 850, and 1900 MHz, respectively. The results are shown in Figure 12a–c for each frequency.

**Figure 13:**
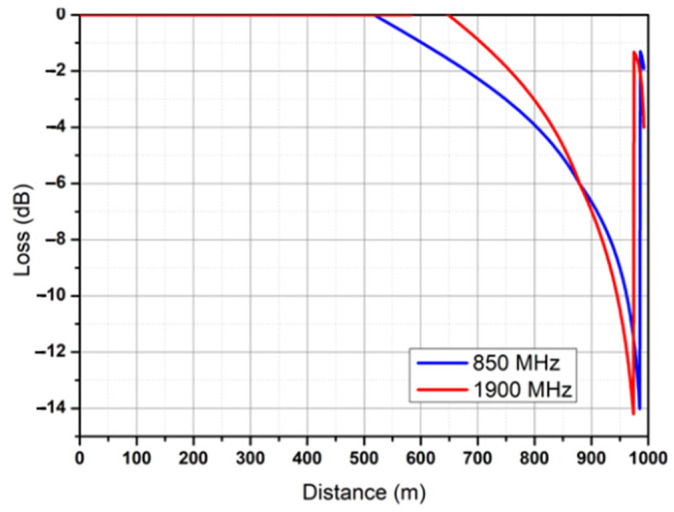
Attenuation due to diffraction effects.

The region between the Caribbean Sea and the GoM is an oceanic zone characterized by a shallow depth and waves with a maximum amplitude of 2 m most of the time; these factors influence the way in which a wireless signal propagates. This influence is observed when studying the diffraction that a wave can produce for the signals transmitted; this influence is quantified with the Kirchhoff parameter in Equation (7). Notably, the possible attenuation that a 2 m wave will produce between the transmitting and receiving antennas is estimated with this equation, and the results are shown in Figure 13 for the frequencies of 850 and 1900 MHz.

**Figure 14:**
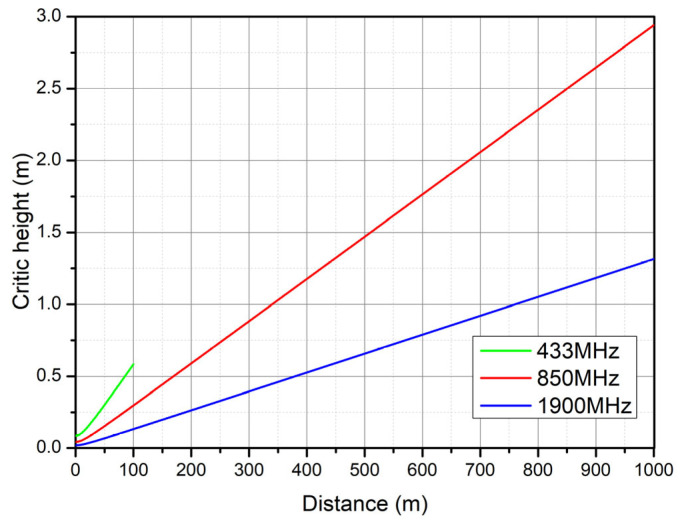
Critical wave height for generating diffuse reflection.

**Figure 15:**
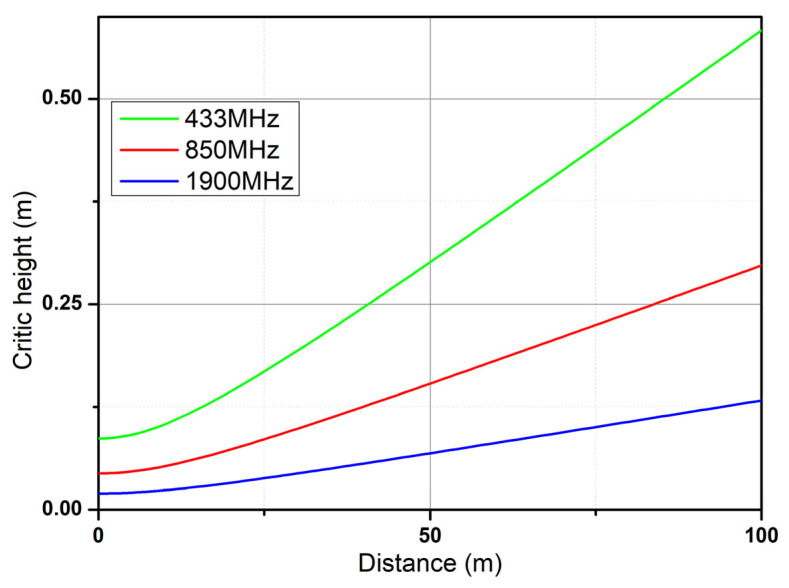
Close-up of the critical height graph.

Irregularities on the water surface can cause a signal to be reflected in a diffuse manner depending on the height of these irregularities in relation to the transmission distance and frequency. Figure 14 shows the height at which the waves produce this phenomenon for the three frequencies, whereas Figure 15 shows a close-up to observe in detail the critical height at the 433 MHz frequency.

### 4.3. Hardware Testing

In this subsection, the results of the energy consumption measurements that were carried out for the system are presented and used to evaluate the autonomy of the system.

To calculate the node’s power consumption, it is first necessary to know its power consumption. This is determined using the following formula [34]:

(10) P=V·I

where V is the node’s supply voltage, and I is the current consumed by the node.

To analyze the energy and power consumption according to the different phases of activity, the following formula is implemented to perform calculations for each activity [34]:

(11) E=P·∆t

where ∆t represents the time, and the node is operating.

**Figure 16:**
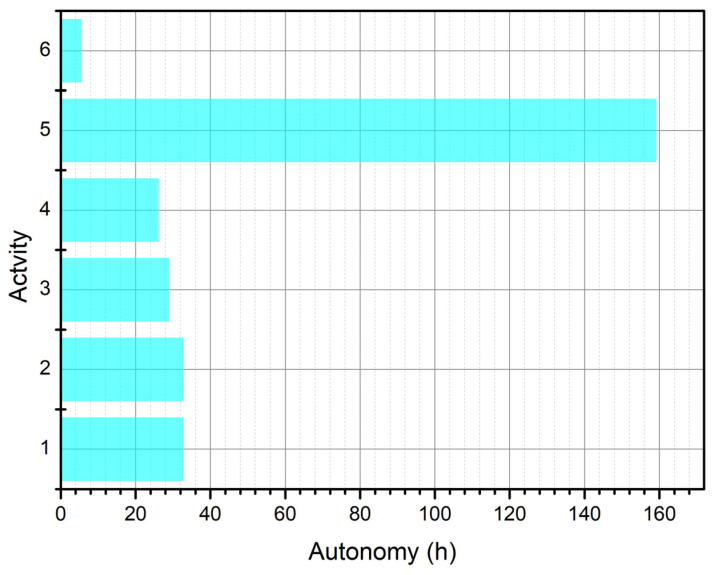
Graph of system autonomy by activity.

However, to obtain a more accurate calculation of energy consumption, it is important to consider the different states of the device: idle and active. In this case, the following formula is used [34]:

(12) $$E=P_{idle}\cdot t_{1}+P_{active}\cdot t_{2}$$

where $P_{idle}$ is the power consumed in the idle state, $P_{active}$ is the power consumed in the active state, $t_{1}$ is the time the device remains idle, and $t_{2}$ is the time the device remains active. Table 2 shows the current and power consumptions values of the system when performing certain activities. In addition, the autonomy of the system was analyzed considering each activity as a unique system element, and the results are shown in Figure 16.

**Figure 17:**
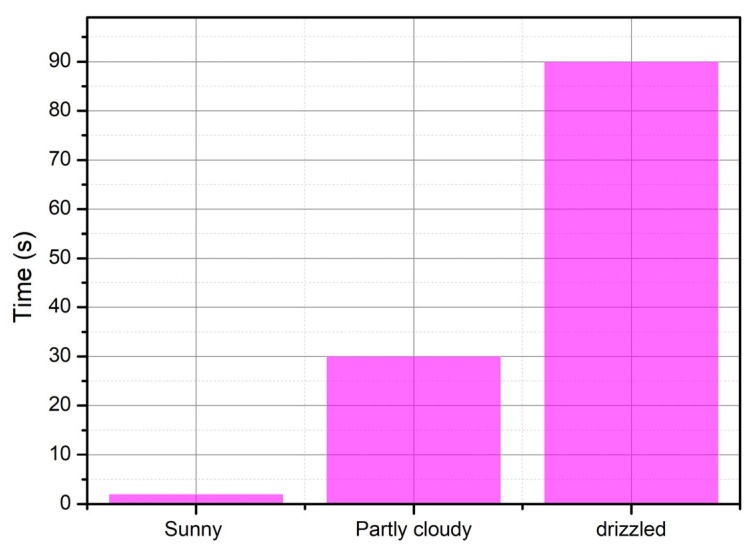
Delay in obtaining a geographic position.

**Figure 18:**
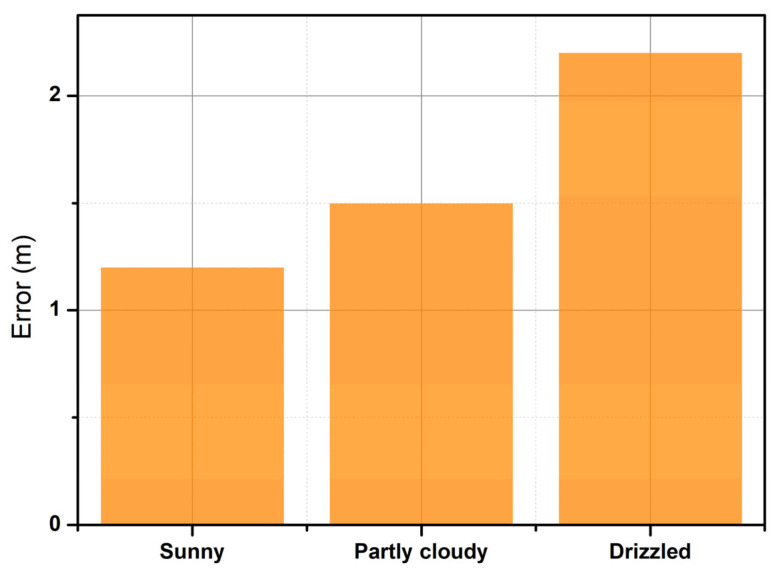
Deviation in geographical position.

Figure 16 shows in blue the autonomy of each activity performed by the node, considering that the average autonomy of the node is 30 h, a period during which the batteries of the system do not need to be recharged. Figure 17 shows in violet the graphs, in three different scenarios, the time it takes for the GPS sensor to capture information about a valid geographic position. Figure 18 shows in orange the graphs, in three different scenarios, the position deviation that the GPS system may present with respect to the actual geographic position.

Below, we show, in Table 3, a comparison of the works analyzed in the section of work related to the results obtained in this work.

From Table 3, we can see the main differences presented in this work. One of our main contributions is working with environmentally friendly technology. We propose working with RF technology, leveraging the wireless communications available on the Mexican coast to track and monitor objects, people, or animals within the coverage area.

### 4.4. Prototype and Hardware Testing

**Figure 19:**
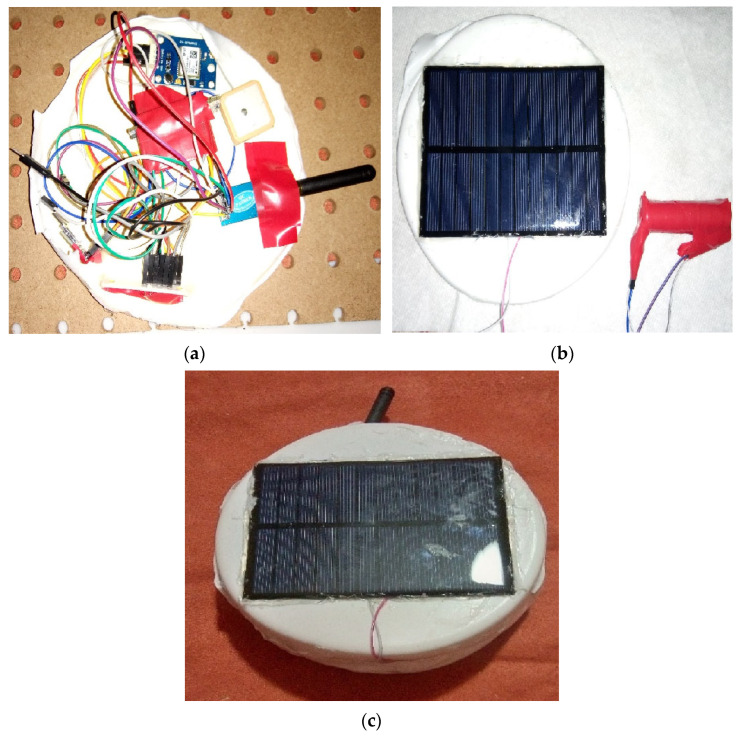
Photographs of the prototype. (**a**) Circuits that make up the sensor node; (**b**) outer cover of the sensor container; (**c**) sealed encapsulation with built-in circuit.

This section shows the built prototype, as well as the results of all the tests performed on the device hardware. Figure 19a shows the interior of the device where the circuits with which the device operates are located. Figure 19b shows the cover of the device where the energy system is located, and Figure 19c shows the fully encapsulated device.

## 5. Discussion and Conclusions

In this research, underwater data transmission via 2.5 G and 3 G cellular communication was analyzed, and it was found that communication channels that utilize low frequencies and minimize attenuation are most effective in this medium. One of the factors that must be accounted for in this environment is wave generation, since these waves limit the LoS, causing the dispersion and diffraction of wireless communication signals. However, based on analyses of the results of diffuse reflection simulations, the dispersion effect can be neglected because the test area is characterized by average wave heights less than one meter. On the other hand, when analyzing diffraction with the Kirchhoff–Fresnel coefficient to determine if there is attenuation due to an intruding wave, it is observed that at a frequency of 1900 MHz, the attenuation is at a minimum, which is a consequence of the thinning of the Fresnel zone. Importantly, the maximum attenuation is 15 dB at the edges of the coverage area proposed in this work, with an effective transmission distance of 1 km for the cellular link and 100 m for the 433 MHz band. The maximum attenuation due to wave diffraction effects is 15 dB at the edges of the 1 km coverage area for the 850 and 1900 MHz bands. We add that the average energy autonomy of the embedded system is 40 h. The embedded system section with the highest power consumption is the cellular link subsystem, with a power consumption of 2 W, which is higher than that provided by the initially selected solar cell.

We conclude that for the 850 MHz frequency, this range is between −46 and −56 dBm, a range 48 levels above the sensitivity of the SARAu201 device. The lower limit values of −63 dBm for 433 MHz and −66 dBm for 850 MHz at 15 cm depth indicate that the electrical properties of seawater directly influence the attenuation.

Finally, the proposed architecture achieves low energy consumption and a high processing capacity, despite the use of a 32-bit system and working frequency limitations. With this architecture, 40 h of autonomous operation can be achieved without the need to recharge the batteries of the system. The highest energy consumption is associated with the wireless communication system, which is related to the transmission power, the type of network card used, and the type of communication.

## Figures and Tables

**Table 1:** Comparison catalog of microcontrollers for the network.

Name	MKR Zero	MSP430F5529 LaunchPad	NUCLEO-L432KC	Curiosity PIC32MZ EF	Quark D2000
Availability	Yes	Yes	Yes	Yes	Yes
Processing bus	32 bits	16 bits	32 bits	32 bits	32 bits
Processor	ARM cortex M0+	MSP430F5529	ARM Cortex M4	PIC32MZ2048EFM100	Quark D2000
Manufacturer	ATMEL	Texas Instrument	STMicroelectronics	Microchip	Intel
Velocity	48–96 MHz	25 MHz	8 MHz–188 MHz	50–200 MHz	32 MHz
Consumption	3.3 V @ 350 mA	1.8–3.6 V @ 10.5 mA	3.3 V @ 144 mA	2.2–3.6 V @ 200 mA	3.3 V @ 84 mA
Serial protocols	SPI, UART, I2C	SPI, UART, I2C, USB	SPI, UART, I2C, USB	SPI, UART, I2C, USB	SPI, UART, I2C, USB
Price	21.9 (USD)	12.99 (USD)	10.32 (USD)	47 (USD)	14.95 (USD)

**Table 2:** Energy consumption of nodes by activity.

Number	Activity	Current Consumption (mA)	Power Consumption (mW)
1	GPS module power on	97.5 ± 3	321.7 ± 10
2	Validating geographic location	97.5 ± 3	321.7 ± 10
3	433 MHz module power-up	110.4 ± 2	364.3 ± 6
4	Transmission of information between nodes	122.3 ± 3	403.59 ± 10
5	Node waiting for a message	20.1 ± 1	66.33 ± 3
6	Internet connection via a cellular link	575 ± 5	1897.5 ±17

**Table 3:** Comparison of the works related to the proposed work.

Work	This Paper	[7]	[8]	[9]	[11]
**Propagation method**	RF	RF	Optical	Acoustic	RF
**Transmission frequency**	433 MHz, 850 MHz and 1900 MHz	2.4 GHz	No data, it is just a proposal	24–28 kHz	433 MHz and 2.4 GHz
**Effective transmission distance (m)**	100 to 1000	500 to 1700	No data, it is just a proposal	No data	0.4
**Energy consumption analysis**	YES	NO	No data, it is just a proposal	No analysis	NO

## Data Availability

The original contributions presented in this study are included in the article. Further inquiries can be directed to the corresponding author(s).
